# Bi-Dimensional Approach Based on Transfer Learning for Alcoholism Pre-disposition Classification via EEG Signals

**DOI:** 10.3389/fnhum.2020.00365

**Published:** 2020-09-18

**Authors:** Hongyi Zhang, Francisco H. S. Silva, Elene F. Ohata, Aldisio G. Medeiros, Pedro P. Rebouças Filho

**Affiliations:** ^1^School of Opto-Electronic and Communication Engineering, Xiamen University of Technology, Xiamen, China; ^2^Laboratório de Processamento de Imagens, Sinais e Computação Aplicada, Instituto Federal do Ceará, Fortaleza, Brazil; ^3^Programa de Pós-Graduação em Engenharia de Teleinformática, Universidade Federal do Ceará, Fortaleza, Brazil; ^4^Programa de Pós-Graduação em Ciência da Computação, Instituto Federal do Ceará, Fortaleza, Brazil

**Keywords:** electroencephalogram, alcoholism, convolutional neural network, computer vision, transfer learning

## Abstract

Recent statistics have shown that the main difficulty in detecting alcoholism is the unreliability of the information presented by patients with alcoholism; this factor confusing the early diagnosis and it can reduce the effectiveness of treatment. However, electroencephalogram (EEG) exams can provide more reliable data for analysis of this behavior. This paper proposes a new approach for the automatic diagnosis of patients with alcoholism and introduces an analysis of the EEG signals from a two-dimensional perspective according to changes in the neural activity, highlighting the influence of high and low-frequency signals. This approach uses a two-dimensional feature extraction method, as well as the application of recent Computer Vision (CV) techniques, such as Transfer Learning with Convolutional Neural Networks (CNN). The methodology to evaluate our proposal used 21 combinations of the traditional classification methods and 84 combinations of recent CNN architectures used as feature extractors combined with the following classical classifiers: Gaussian Naive Bayes, K-Nearest Neighbor (k-NN), Multilayer Perceptron (MLP), Random Forest (RF) and Support Vector Machine (SVM). CNN MobileNet combined with SVM achieved the best results in Accuracy (95.33%), Precision (95.68%), F1-Score (95.24%), and Recall (95.00%). This combination outperformed the traditional methods by up to 8%. Thus, this approach is applicable as a classification stage for computer-aided diagnoses, useful for the triage of patients, and clinical support for the early diagnosis of this disease.

## 1. Introduction

In 2016, there were around 3 million deaths worldwide due to alcohol abuse, 5.3% of all deaths recorded that year. The number of deaths from alcohol is greater than from some other serious diseases like tuberculosis, Acquired Immunodeficiency Syndrome (AIDS) and diabetes (World Health Organization, 2019). Still in 2016, alcohol caused 132.6 million disability-adjusted life years (DALYs) which represented 5.1% of all DALYs in that year. The World Health Organization (WHO) estimates that 283 million people worldwide have alcohol use disorders (World Health Organization, 2019).

Moderate and frequent alcohol consumption (>30 g/day) can bring benefits to the cardiovascular system (Foppa et al., 2001), with increased High Density Lipoprotein (HDL) cholesterol and the consumption of red wine has antioxidant action (da Luz and Coimbra, 2004). However, abusive alcohol consumption (>60 g/day) has direct consequences for the medium-long term health of the individual, such as liver disease, cancer, cardiovascular, and mental problems, as well as indirect consequences in case of accidents, suicides, and homicides due to short-term harm, such as cognitive and mobility problems (da Luz and Coimbra, 2004; Jennison, 2004; World Health Organization, 2019). Alcohol affects the Central Nervous System (CNS) directly, causing changes in its function and in brain functions. One way to check brain activity and the changes caused by alcohol is through an EEG exam (Devor and Cloninger, 1989) which can identify different types of brain activities through electrodes placed on specific regions of the head.

The EEG has multiple channels to collect electrical signals, which are emitted through neuron synapses and are incredibly complicated and non-linear. Specific techniques are required to interpret the complexity of these exams. Computer Aided Diagnostic (CAD) tools along with the use of Digital Signal Processing (DSP) and Artificial Intelligence (AI) techniques, with Machine Learning (ML) (Acharya et al., 2012; McBride et al., 2015; Patidar et al., 2017; Bhattacharyya et al., 2018; Bosl et al., 2018; Ibrahim et al., 2018; Amezquita-Sanchez et al., 2019; Rodrigues et al., 2019) can be applied to interpret these signals. Several studies have been performed using EEG signals to identify different types of disturbances in brain activity, such as the detection of patterns that characterize Alzheimer's disease (McBride et al., 2015; Amezquita-Sanchez et al., 2019; Tzimourta et al., 2019), autism (Boutros et al., 2015; Bosl et al., 2018; Ibrahim et al., 2018), sleep disorders (Koley and Dey, 2012; D'Rozario et al., 2015; Rundo et al., 2019), hyperactivity (Mohammadi et al., 2016; Muñoz-Organero et al., 2018; Wang et al., 2019), and epilepsy (Bhattacharyya et al., 2018; Ibrahim et al., 2018; Ren et al., 2018).

Besides the fact that EEG exams have previously presented good results in identifying different diseases, we chose to use the EEG exam because it provides an extensive mapping of brain activity equal to other exams, such as Magnetoencephalography (MEG), functional Magnetic Resonance Imaging (fMRI), functional Near-Infrared Spectroscopy (fNIRS) and Positron Emission Tomography (PET). However, recording EEG signals is simpler than MEG signals, since the measurement of electrical voltages is more easily performed than the measurement of magnetic fields as they have a low amplitude (Stam, 2010). Hair artifacts can influence infrared-based fNIRS measurements, and thus directly interfering with the reliability of the exam (Lloyd-Fox et al., 2010). EEG does not emit particles to obtain the result of the examination, as in the case of PET (Chugani et al., 1987). Furthermore, fMRI (Kozel et al., 2004) requires the use of high-cost magnetic scanners unlike EEG, which in comparison is a low-cost equivalent solution.

### 1.1. Contribution and Paper Organization

Among the main contributions of this work to diagnose a predisposition to alcoholism, we highlight the use of a heat map to represent the brain activity of each patient in order to provide a visual analysis and the use of the Transfer Learning method, as the extraction of deep attributes as a way to represent the healthy and pathologic samples.

The paper is organized as follows: section 2 presents a literature review concerning the topic. Section 3 discusses the materials and methods that support the proposed technique. Section 4 gives a description of the use of CNN as an attribute extractor. The proposed methodology is described in section 5, and finally, in section 6, we present the results obtained and the discussion.

## 2. Overview of the Alcoholism Predisposition Classification

This section presents the state of the art of EEG analysis to identify alcoholism considering the evolution of feature extraction methods from the traditional statistical approach to the current use of CNNs as feature extractors.

Acharya et al. (2012) developed an automatic technique for CAD to identify healthy patients with a genetic predisposition to alcoholism through EEG signal analyses. These authors combined a non-linear feature extraction, such as Approximate Entropy, Sample Entropy, Largest Lyapunov Exponent, and four Higher-Order Spectra (HOS) functions with a SVM classifier, varied the Polynomial and Radial Basis Function (RBF) kernals. Their results indicated that non-linear measurements extracted from EEG signals can achieve promising results.

Using the electrical impulses that represent the physiological functions like eye blinking and heart beating, Rachman et al. (2016) proposed an independent component analysis through EEG signals. In their work, the features extracted by stationary wavelet transform with Daubechies decomposition at level 6 were combined with a probabilistic neural network to classify samples from 64 channels into two classes: healthy and alcoholism patients. However, this work only used classical statistic features like maximum, minimum, and average values, showing its fragility when outlier samples were present in the dataset.

Mumtaz et al. (2016), on the other hand, analyzed 19 channels placed according to the international 10–20 system to identify healthy and alcoholism patient. The dataset had 18 alcoholism and 15 healthy patients. They extracted features through quantitative electroencephalography from EEG data. The features were used as the input for classification models: Linear Discriminant Analysis, SVM, MLP, and Logistic Model Trees. This study suggests that EEG spectral analysis can help to classify pathologic samples from the healthy ones. Nevertheless, they used seven frequency bands in these analyses, indicating an increase in the time to generate results.

Ehlers et al. (1998) proposed an approach to evaluate the influence of alcohol consumed on brain activities. They analyzed EEG signals through temporal series combined with the chaos theory. In their study, the authors assessed two groups of patients, a control group, and an alcoholism group. Based on this, they suggested that the EEG signal has non-linear structures that can be modified when the patient is under the effect of alcohol.

Kannathal et al. (2005) analyzed EEG signals through non-linear measurements, such as correlation dimension, largest Lyapunov exponent, Hurst exponent, and entropy values. The authors suggested that non-linear analysis could contribute to distinguish between healthy and alcoholic patients.

Faust et al. (2013) also considered the non-linear characteristics of EEG signals. These authors used the non-linear feature of HOS to extract information about alcoholic patients. This feature was used as the input to six different classifications models: Decision Tree, Fuzzy Sugeno Classifier, k-NN, Gaussian Mixture Model, Naive Bayes Classifier, and Probabilistic Neural Network.

Although these recent works in the literature have presented promising results, some of them omitted the number of samples evaluated and which criteria was used to select the channels of the EEG exam to be analyzed. Furthermore, most of these works use feature extraction techniques specially adjusted to assessed datasets, hindering the possibility to generalize to signals with other characteristics. Finally, these works did not evaluate new feature extractors, especially such algorithms based on the recent technique of Deep Learning (DL) using Transfer Learning; this is one of the innovations of our approach.

Moreover, these works were performed using the raw one-dimensional signals of the EEG, in addition to selecting specific channels to solve the problem. In our work, we proposed a two-dimensional heat-map representations to represent the EEG channels, where each value acquired from one channel corresponds to the pixel value in the resulting image, so the junction of all selected channels makes up the final image for each patient.

The generated image corresponds to the heat map of the brain activity of this patient, thus giving a visual analysis of the problem, as well as the use of CV, DL, and ML methods. The use of heat map imaging enables the application of structural and textural analysis methods, such as pixel variance, morphological gradient calculations, equalization, as well as enhancement algorithms that can improve the distinction between alcoholic and healthy samples; thus giving a more accurate diagnostic.

The two-dimensional approach also allows the use of feature extraction methods, which describe different shapes, textures and structures of each image, such as Gray-Level Co-Occurrence Matrix (GLCM) (Haralick et al., 1973), Hu's Moments (Hu, 1962), and Local Binary Patterns (LBP) (Ojala et al., 2002). Furthermore, the application of the Transfer Learning technique using CNNs enables the extraction of the most relevant features from an image through extreme non-linear models. The classification of these characteristics belonging to each patient is obtained using ML algorithms. Through a Random Search for the optimal parameters, we obtained the best configuration of the following models: k-NN (Fukunaga and Narendra, 1975), MLP (Haykin, 2008), RF (Breiman, 2001), and SVM (Vapnik, 1998).

## 3. Materials

In this section, we present the digital image processing techniques and the ML that supports the methodology proposed in this work.

### 3.1. Dataset

The dataset used in this work is publicly available in Begleiter (2019) from the University of California, Irvine, and is known as Knowledge Discovery in Database (UCI KDD). This dataset was initially developed to examine genetic predisposition, through EEG signals, to alcoholism. Two subject groups made up the dataset: an alcoholics group and a control group. The Alcoholic group consists of the 77 male subjects with a mean age of 35.83 ± 5.33. The control group consists of 48 male subjects with no particular or family history of alcohol misuse or neurological disorder or any history of psychiatric disease.

The signal acquisition is according to the 10–20 International System with 64 electrodes placed on the scalps of the subjects, with a sampling frequency of 256 samples per second. The Cz electrode is taken as a reference. Each signal has a period of 190 ms of a pre-stimulation and 1,440 ms after each stimulus.

Each subject was exposed to three conditions, a single stimulus (S1) was presented to each subject. A second stimulus (S2) is a matching condition, here the same stimulus S1 was repeated. Finally, the last stimulus (S3) presented in either a matched condition where S1 was identical to S2. Each stimulus corresponds to a picture of objects chosen from the 1980 Snodgrass and Vanderwart picture set (Snodgrass and Vanderwart, 1980).

### 3.2. Tradictional Feature Extraction Methods

In this study, three feature extraction methods were used to improve the analysis of the proposed approach.

Haralick et al. (1973) proposed a statistical analysis considering the co-occurrence of gray levels in the image. This method is called Gray-Level Co-Occurrence Matrix (GLCM) and identifies the spatial influences of pixels related to their grayscale. GLCM has 14 features, and among which the angular second moment and entropy are commonly used and here they are presented in Equations (1) and (2), respectively, where *p* is central pixel, *i* and *j* are indexes according to image height and width.

(1) $$\begin{array}{r} \underset{i}{\sum }\underset{j}{\sum }p{\left(i,j\right)}^{2} \end{array}$$

(2) $$\begin{array}{r} -\underset{i}{\sum }\underset{j}{\sum }p\left(i,j\right)log\left(p\left(i,j\right)\right) \end{array}$$

The Local Binary Patterns (LBP) proposed by Ojala et al. (1994), was developed as an efficient and straightforward way to describe the texture of an image. LBP extracts information from the local gray scale levels of the image to define a pattern that represents P pixels of the near neighbors. This binary pattern follows a pattern determined by neighbors analysis direction. Equation (3) presents the neighborhood analysis, where *g*_*p*_ is a neighbor pixel *P* to the region of the radius *R*, and *g*_*c*_ is the central pixel.

(3) $$\begin{array}{r} LBP_{P,R}=\overset{P-1}{\underset{p=0}{\sum }}f{\left(g_{p}-g_{c}\right)}^{2P} \end{array}$$

According to the threshold *x*, a binary pattern is assigned to each operation (Equation 4).

(4) $$\begin{array}{r} f\left(x\right)=\{\begin{array}{ccc} 1, & if & x\geq 0\\ 0 & otherwise \end{array} \end{array}$$

Hu (1962) developed a model that uses central moments to make the method invariant to scale and rotational changes. This method, known as HU moments, describes a feature extraction family composed of seven moments; each one is invariable to size, rotation, and translation operations. Equation (5) shows the relation between central moment and normalized moment. This normalized moment can be obtained from the central moment, μ_*pq*_, divided by an exponential of the area, μ_00_, to obtain the normalized central moment, η_*pq*_.

(5) $$\begin{array}{r} \eta_{pq}=\frac{\mu_{pq}}{\mu_{00}^{\alpha }} \end{array}$$

where

(6) $$\begin{array}{r} \alpha =\frac{p+q}{2};\forall p+q\geq 2 \end{array}$$

### 3.3. Classifiers

This section describes the ML techniques used to classify the features extracted by the traditional methods and the CNN architectures.

#### 3.3.1. Naive Bayes

Bayesian Classifier is based on statistical analysis of input data. The classifications are based on the probability distribution of each sample to a specific class, considering that this class has the highest probability to be associated with the sample (Theodoridis and Koutroumbas, 2008). The Bayes Theory inspires this model, and it assumes that there are no dependencies among the features, according to the value of posterior probability and conditional probability.

#### 3.3.2. K-Nearest Neighbor

K-Nearest Neighbor (k-NN) is a machine learning method proposed by Fukunaga and Narendra (1975) that falls into the supervised category. It determines the class to which a sample belongs by comparing the features of the *k* nearest neighbors that were acquired in a previous training step. The variable *k* represents the number of samples of the training set that possess the closest features to the sample being classified. Still regarding the variable *k*, there is not a standard value for it, but in general, even numbers are avoided to prevent a drawn situation in which the sample could be classified into two classes at the same time.

#### 3.3.3. Multilayer Perceptron—MLP

Multilayer Perceptron (MLP) is a neural network architecture formed by multiple layers with perceptron neurons. The input data vector is introduced to the first layer where each feature is computed and each neuron contributes to transform the input space into a linearly separable space and thus to classify the object in its specific class (Haykin, 2008). The learning technique is supervised through a backpropagation algorithm where the errors calculated at the last layer are retro propagated to adjust the hidden layers (Haykin, 2008). Therefore, throughout this procedure, the solution to samples in the input vector is presented in the output layer.

#### 3.3.4. Random Forest

Random Forest (RF) is based on decision trees, proposed by Breiman (2001). It aims to make a decision tree using a set of features selected from the initial set. The training is achieved by using a meta-algorithm called bagging, which uses the stability and accuracy of the results to improve the classification. Bagging is used to reduce the variance and over-fitting. After the tree sets are created, it is possible to determine which set contains the best configuration to solve a problem.

#### 3.3.5. Support Vector Machine—SVM

A Support Vector Machine (SVM) is based on the statistic distribution of the samples in the input vector proposed by Suykens and Vandewalle (1999). SVMs aim to identify samples that are most difficult to classify because they are close to the decision boundary. This method uses the optimization theory to adjust the optimal decision boundary for the minimization of the cost function with restriction parameters. Originally developed for binary classification, this classifier can be extended to multiclass problems through the one-against-all and one-against-one approaches, in addition these are techniques based on the graph theory (Vapnik, 1998). SVMs can be applied to both linear and non-linear problems, this latter method can use an RBF type kernel.

## 4. Convolutional Neural Networks

In this paper, we evaluated the following CNNs: DenseNet (Huang et al., 2017), Inception-ResNet (Längkvist et al., 2014), Inception (Szegedy et al., 2015), MobileNet (Howard et al., 2017), NasNet (Zoph and Le, 2016), ResNet (Wu et al., 2018), VGG (Simonyan and Zisserman, 2014), and Xception (Chollet, 2017).

### 4.1. Convolutional Neural Networks as Feature Extractor

In this paper, CNNs used the transfer learning concept, which relates the descriptive power of a pre-trained CNN on samples of a problem not yet known by the model. The first fully connected layer is removed, and then, a resizing of its input is transformed into a one-dimensional array. After this process, a pre-trained model does not behave as a classifier, so it is used as a feature extractor. The transfer learning technique is detailed in the work of da Nóbrega et al. (2018), who applied transfer learning to lung nodule classification.

#### 4.1.1. Architecture Construction and Initialization

Many architectures have been proposed in the last few years, especially since 2010, with the advent of object recognition challenges in large scale image datasets (Deng et al., 2009). However, it is not viable to evaluate all of the architectures proposed by the scientific community; therefore, 12 well-known architectures were selected for the experiments of this work. The configurations of the models described in their respective paper were used during implementation.

#### 4.1.2. Architecture Training

The twelve architectures were trained from the ImageNet dataset (Deng et al., 2009), which consists of 1.2 million non-medical images, and grouped into 1,000 categories. The training methodologies used by each architecture are documented in detail in their respective articles. This step was done based on the premise that the features learned by a CNN to discriminate a set of classes are capable of representing other samples as the model can extrapolate the known patterns to new sample with the use of transfer learning technique.

#### 4.1.3. Converting CNNs Into Feature Extractor

In this last step, the CNNs trained on the previously mentioned set are transformed into feature extractors. Nonetheless, to perform this step, it is crucial to understand the four transformations executed by these neural networks.

Initially, the input image is submitted to a sequence of non-linear transformations. These transformations are defined depending on the architecture used. In this first stage, the input image is converted into a set of small matrices. Secondly, each of these matrices is resized to a one-dimensional array. Then, the set of arrays is concatenated, thus generating a single array. Each one-dimensional array can be interpreted as a feature vector that represents the heat map image. Lastly, the features vectors are submitted to a classifier training. With the modified architecture, the results of the model should not be interpreted as a probability set of an input image related to a determined label but should be interpreted as an information vector, which will be used by an external classifier to compose the probabilities of predisposition to alcoholism.

**Figure 1:**
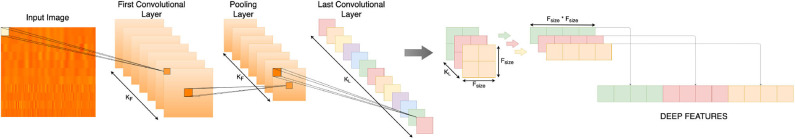
Transfer learning figure.

Figure 1 shows the fully connected layer after the removal of the last convolutional layer. The outputs are concatenated and then the vectors set that will be used to train and test the classifier are created.

## 5. Methodology

**Figure 2:**
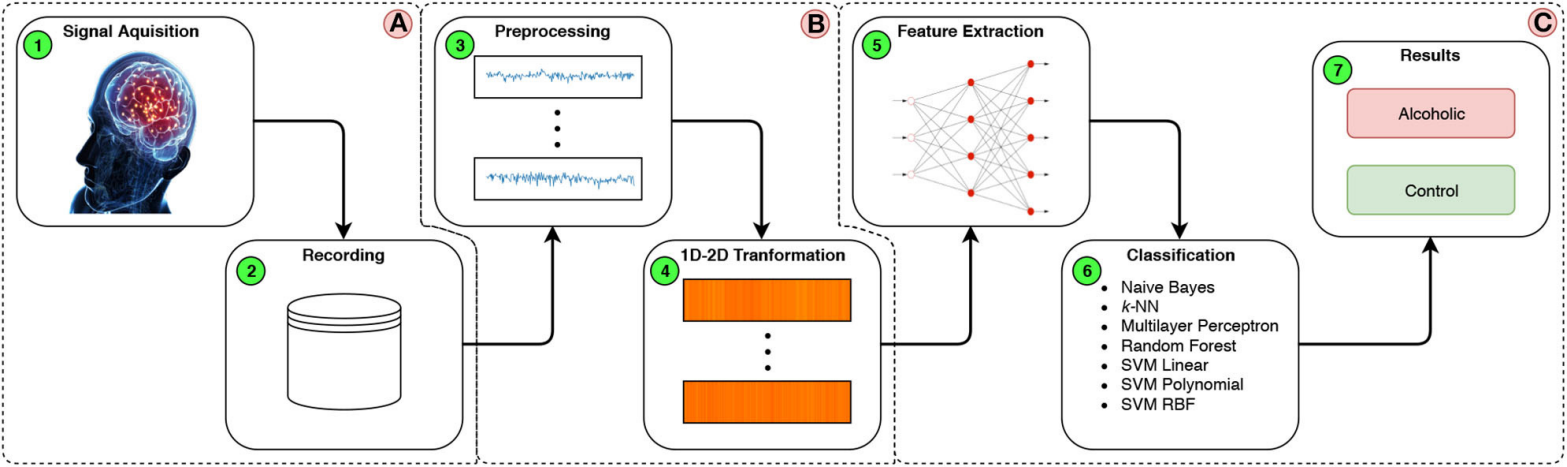
Flow chart of the proposed methodology.

In this paper, we propose the detection of a predisposition to alcoholism comparing EEG signals from two subgroups: alcoholism and control. Figure 2 illustrates the proposed methodology step-by-step and it is divided into three main stages: Acquisition of the EEG signals (A), Digital Signal Processing (DSP) (B), and finally, extraction and classification of the samples (C).

### 5.1. Pre-processing Step

Out of the 64 exam channels, only 11 were selected from the mean-variance of each channel for all patients in the dataset. The selected channels were: FP1, FP2, F7, F8, T8, T7, T7, CZ, C3, C4, CP5, and CP6. These channels presented the highest values of variance in their signals, which means more intense brain activity in the regions where these channels were located.

**Figure 3:**
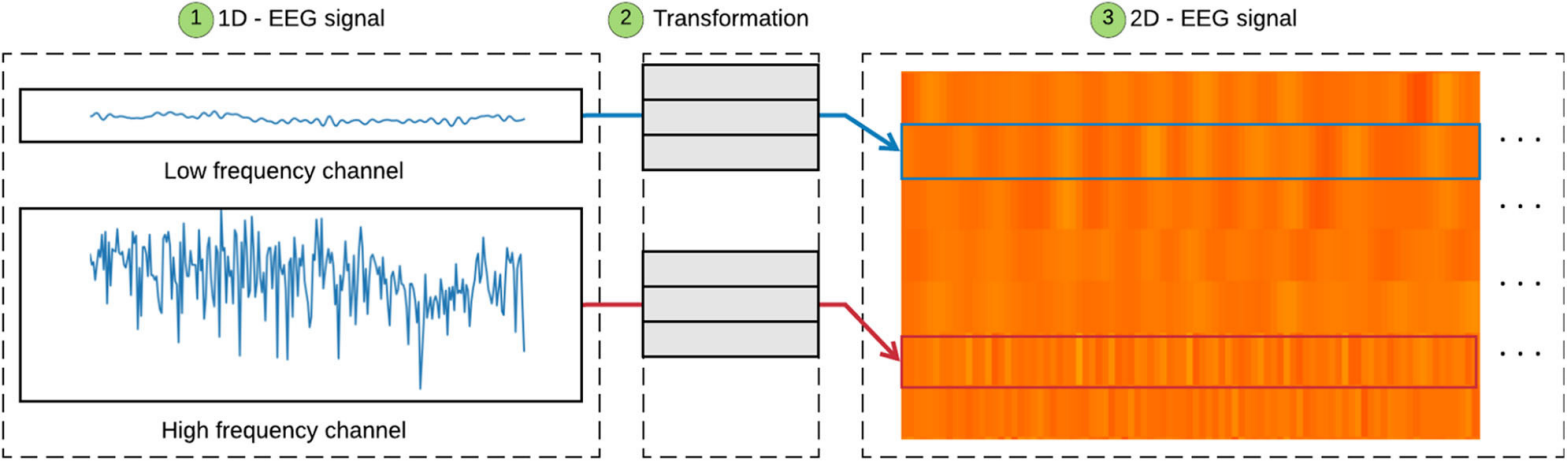
The transformation from 1D EEG signals to 2D EEG signals. Low-frequency channels are transformed to a smooth texture block. High-frequency channels are transformed to a rough texture block.

**Figure 4:**
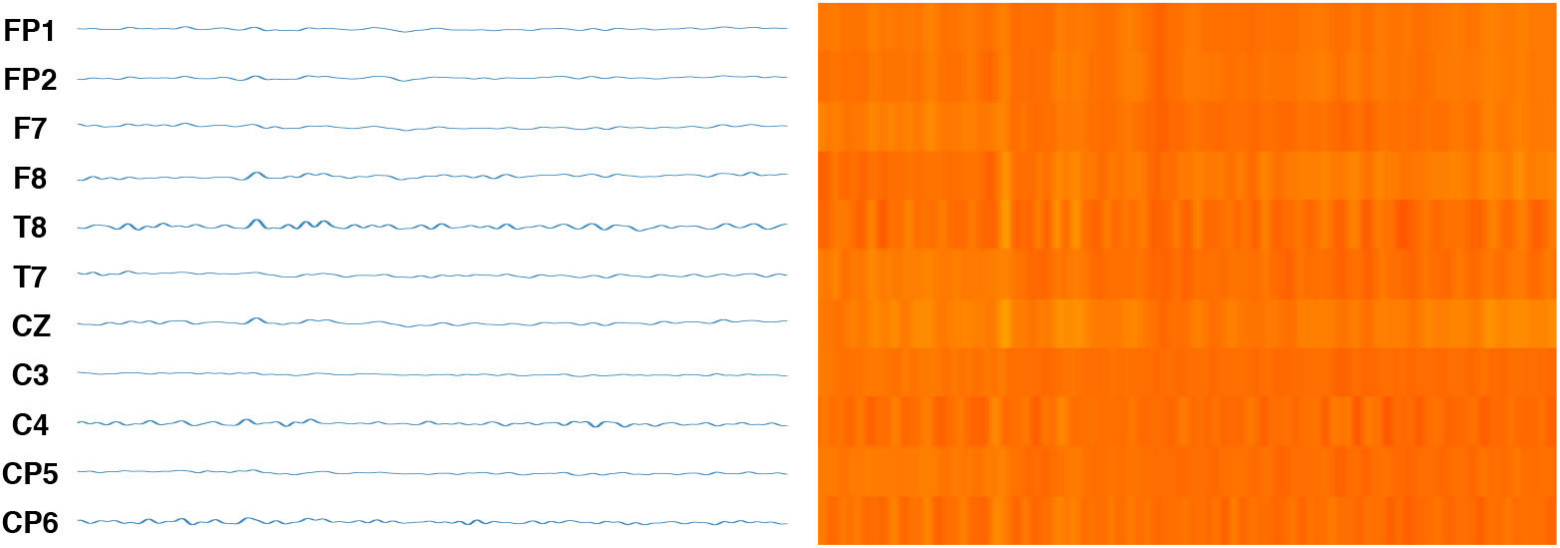
On the left, original EEG signals. On the right the final image after channels transformation from the 1D channels to a 2D image.

Initially, stage A was performed out during the formation of the Dataset. In stage B the data is prepared in step 3 (Figure 2B-3) by removing any outliers, >73.3 and < −73.3 uV, which represent possible head and eye movements (Zhang et al., 1995), and then the set of signals is normalized within a range of 0–1. In step 4 (Figure 2B-4), the interval is readjusted to 0–255, in addition to turning all values into integers, which enables the creation of an 8-bit image with 1,024 × 352 shape that represents the concatenation of the exam channels, where each of the selected channels are 1,024 × 32 pixel regions, as shown in Figure 3.

Finally, in stage C, the CNN technique is used as a feature extractor combined with a Transfer Learning method. The extracted features are classified in Alcoholic and Control, using traditional classification models.

Figure 3 shows the transformation process from a 1D EEG signal to a 2D EEG signal, highlighting the distinction between low and high-frequency. Step 1 (Figure 3-1) shows the 1D EEG signals. The channels are transformed into a 2D image in step 2 (Figure 3-2) as previously described. Step 3 (Figure 3-3) shows that high-frequency signals are represented by roughly textured blocks, creating a surface with peaks and valleys due to the high variation of the signals. On the other hand, low-frequency signals are represented by smooth texture blocks, presenting a flat surface due to the low variation of the signals. We found such signals, high and low frequency, using the calculation of the mean-variance of the exam channels.

Finally, Figure 4 shows a sample of the complete transformation from the 1D channels to a 2D image. This approach becomes a visual representation of brain activity in different parts of the brain, rather than treating each channel separately. This image corresponds to a heat map of the brain activity in the regions measured by the electrodes. This image clearly represents the variations of time-domain and reflect the temporal variations of the channels through image texture, as well as the EEG signal by the intensity of color. This approach permits a visual analysis of the problem, as well as the use of structural and textural analytical methods. Moreover, the approach makes it possible to use recent methods of CV, DL, and ML methods.

### 5.2. Feature Extraction

**Figure 5:**
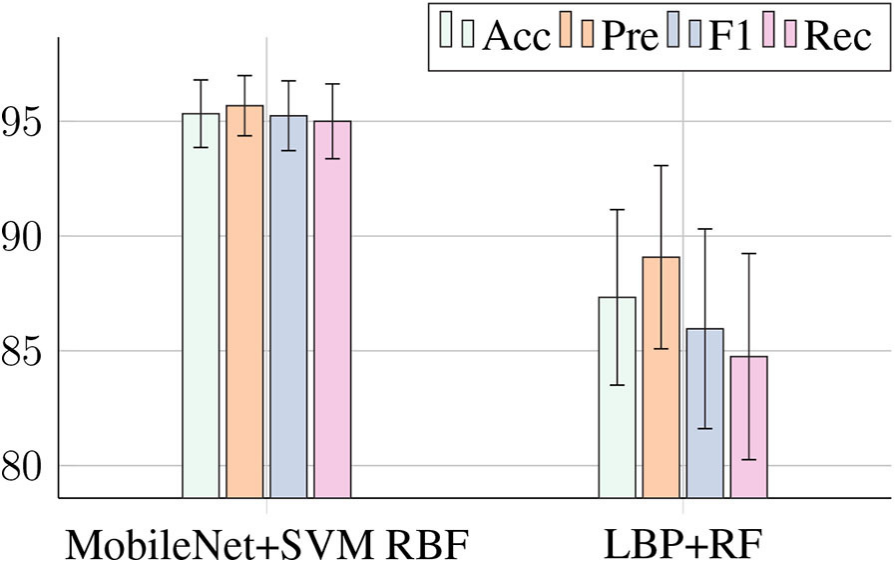
A plot representation of the best metrics from Tables 2–4.

In Figure 2 stage 2-C, step 5 (Figure 2C-5), according to section 4.1.3, this approach proposes the use of CNN techniques as a feature extractor combined with a Transfer Learning method for the two-dimensional signals. The image that represents the EEG signals is processed by the first convolutional layers of the neural network, and the output of the final layer of the CNN is combined as a feature vector for the classification stage. The traditional computational vision extractors, such as GLCM, Hu Moments, and LBP were evaluated. Table 1 shows the number of features generated by each extractor.

**Table 1:** Number of features returned by each extractor.

**Approach**	**Extractor**	**Number of features**
Traditional	LBP	48
	GLCM	14
	Hu Moments	7
Transfer Learning	Densenet 121	1,024
	Densenet 169	1,664
	Densenet 201	1,920
	InceptionResNetV2	1,536
	InceptionV3	2,048
	MobileNet	1,024
	NASNetLarge	4,032
	NASNetMobile	1,056
	ResNet50	2,048
	VGG16	512
	VGG19	512
	Xception	2,048

### 5.3. Classification Healthy and Alcoholism Patient

To evaluate the representativeness of the extracted features for the classification of both sets, healthy patients and alcoholic patients, the generated dataset is classified using five consolidated ML techniques: Bayes (Theodoridis and Koutroumbas, 2008), k-NN (Fukunaga and Narendra, 1975), RF (Breiman, 2001), MLP (Haykin, 2008), and SVM (Vapnik, 1998).

In the classification process, Bayes classifier operated with the Probability Density Function (PDF). MLP performed its training using the Levenberg-Marquardt method, and with the neurons varying from 2 to 1,000 in the hidden layer. The number of neighbors for the k-NN classifier was determined through a grid search, where the *k* value was varied using the odd values from 3 to 15.

The SVM classifier used linear, polynomial and RBF kernels. In all three configurations, the *C* hyperparameter was defined as 2^−5^, 2^−4^, 2^−3^, …, 2^15^. For the RBF kernel, γ was varied from 2^−15^ to 2^3^, while for polynomial kernel, the degree ranged using the odd values from 3 to 9.

For the RF classifier, the criteria function was varied for Gini and entropy, the minimum number of samples that is necessary to split an internal node ranged from 1 to 6, the lowest amount of samples requested to be at a leaf node also ranged from 1 to 6, and the number of estimators was 3,000.

The training stage of the classification models considered the cross-validation technique. Of the total samples, 77 represent patients in the Alcoholic group, and 48 represent the control group. The samples were divided into ten subsets with a proportion of 80% for training and 20% for tests, randomly chosen. The hyperparameters for MLP, SVM and RF were determined through a 20-iterations random search over a cross-validation process with 10-folds.

The classification stage completes the C stage of the proposed methodology. The evaluation metrics and results are discussed below.

### 5.4. Evaluation Metrics

To compare our classification results with results from other methods, we use evaluation metrics based on the results obtained in the confusion matrix. The results of the confusion matrix include True Positive (TP), True Negative (TN), False Positive (FP), and False Negative (FN), some of which were used for the evaluation metrics. The evaluation of this approach used the following metrics:

Accuracy (Acc) (Fawcett, 2006) reveals the proximity of the result to the gold standard and is given by the relationship between the hits and the set of all predictions, and is presented by Equation (7).

(7) $$\begin{array}{r} Acc=\frac{TP+TN}{TP+TN+FP+FN} \end{array}$$

The value of Precision (Fawcett, 2006) is the probability of true positives relative to all results classified as positive and is presented by Equation (8). Even if the test diagnosis is positive, this metric calculates the probability that the test will be consistent with the prior probability.

(8) $$\begin{array}{r} Precision=\frac{TP}{TP+FP} \end{array}$$

Recall (Rec) (Sokolova and Lapalme, 2009) represents the proportion of the results classified as positive among all the results that are really positive and is presented by Equation (9).

(9) $$\begin{array}{r} Recall=\frac{TP}{TP+FN} \end{array}$$

As a counterpoint to Precision, considering its risk of imbalance, the F1 Score () calculates the weighted harmonic mean between Precision and Recall and is presented by Equation (10). The F1 Score represents the performance of a method and although a diagnosis may be classified accurately, it does not mean that the method will perform the same for other data.

(10) $$\begin{array}{r} F1Score=\frac{2*Rec*Precision}{Rec+Precision} \end{array}$$

Except for the F1 Score index, all other evaluation measures were investigated in previous studies with signs of EEG (Ehlers et al., 1998; Acharya et al., 2012; Mumtaz et al., 2016; Rachman et al., 2016; Patidar et al., 2017).

## 6. Results and Discussion

The proposed approach was evaluated on a computer with an Intel Core i7 microprocessor, 8 GB of RAM, a GeForce GTX 1070 Graphics Processing Unit (GPU), and a Linux LTS 16.04 operating system. The results of this paper are presented in three stages. In the first stage, the evaluation of the 21 combinations of the traditional methods for image feature extraction and classifiers. The second stage is the evaluation of the 84 combinations of CNNs as the feature extractors and classifiers. Finally, the best results are compared to related works in the last stage.

Average values and standard deviations of Accuracy, F1-Score, Precision, and Recall are shown in Tables 2–4 for the features extracted with traditional methods and CNN-based methods, respectively.

**Table 2:** Accuracy, Precision, F1-Score, and Recall obtained through the classification of extracted features with classical extractors.

**Extractors**	**Classifiers**	**Accuracy**	**Precision**	**F1 Score**	**Recall**
GLCM	Naive Bayes	64.44 ± 1.22	81.61 ± 0.40	46.55 ± 2.96	54.29 ± 1.56
	MLP	64.44 ± 1.22	81.61 ± 0.40	46.55 ± 2.96	54.29 ± 1.56
	kNN	86.11 ± 3.23	87.70 ± 3.97	84.66 ± 3.69	83.55 ± 3.73
	**RF**	**87.22** **±** **2.87**	**89.06** **±** **3.40**	**85.86** **±** **3.18**	**84.56** **±** **3.12**
	SVM Linear	73.22 ± 3.12	78.87 ± 4.39	66.47 ± 4.96	66.71 ± 4.05
	SVM Polynomial	64.44 ± 1.22	81.61 ± 0.40	46.55 ± 2.96	54.29 ± 1.56
	SVM RBF	72.22 ± 2.77	84.11 ± 1.14	62.83 ± 4.91	64.34 ± 3.62
HU	Naive Bayes	61.11 ± 0.00	30.56 ± 0.00	37.93 ± 0.00	50.00 ± 0.00
	MLP	61.11 ± 0.00	30.56 ± 0.00	37.93 ± 0.00	50.00 ± 0.00
	kNN	80.44 ± 3.19	81.64 ± 4.05	78.14 ± 3.85	77.19 ± 3.74
	**RF**	**80.67** **±** **3.30**	**81.59** **±** **4.08**	**78.51** **±** **3.82**	**77.58** **±** **3.78**
	SVM Linear	52.89 ± 3.79	55.34 ± 3.52	52.81 ± 3.78	55.38 ± 3.64
	SVM Polynomial	51.89 ± 2.77	55.72 ± 2.91	51.75 ± 2.87	55.44 ± 2.84
	SVM RBF	50.56 ± 4.28	57.97 ± 3.80	49.31 ± 5.34	56.17 ± 3.54
LBP	Naive Bayes	61.11 ± 0.00	30.56 ± 0.00	37.93 ± 0.00	50.00 ± 0.00
	MLP	61.11 ± 0.00	30.56 ± 0.00	37.93 ± 0.00	50.00 ± 0.00
	kNN	83.89 ± 2.87	84.81 ± 4.13	82.41 ± 2.89	81.47 ± 2.64
	**RF**	**87.33** **±** **3.82**	**89.08** **±** **3.99**	**85.96** **±** **4.35**	**84.75** **±** **4.49**
	SVM Linear	66.89 ± 3.33	65.00 ± 3.94	63.21 ± 3.81	63.09 ± 3.55
	SVM Polynomial	38.89 ± 0.00	19.44 ± 0.00	28.00 ± 0.00	50.00 ± 0.00
	SVM RBF	68.56 ± 2.33	71.01 ± 5.78	60.57 ± 3.66	61.70 ± 2.77

*The bold values are mean and standard deviation, respectively*.

Analyzing Table 2, GLCM-k-NN, GLCM-RF, HU-k-NN, HU-RF, LBP-k-NN, and LBP-RF stand out as they achieved at least 80% in Accuracy. Also, the RF classifier can be highlighted, since it achieved the highest Accuracy when combined with all three traditional methods. The best combination (LBP-RF) is highlighted in green. This combination reached the highest values in all four metrics.

Tables 3, 4 show the results of features extracted using CNNs, and then classified. The combinations that achieved a minimum of 90% of Accuracy and Recall were: MobileNet-k-NN, MobileNet-SVM Linear, MobileNet-SVM Polynomial, MobileNet-SVM RBF, NasNetMobile-SVM RBF, VGG16-SVM RBF, VGG19-k-NN, VGG19-SVM RBF, and Xception-SVM RBF. The combinations that had at least 90% in Accuracy, but did not achieve this value in Recall were disregarded since low values of Recall are not desirable in order not to classify alcoholism as healthy. The SVM classifier stands out when classifying deep features. This classifier obtained the best metrics values for all CNN extractors, except for ResNet50, in which the best classifier was k-NN. Among the SVM kernels, RBF reached the highest metric values for ten of the twelve CNN architectures evaluated. The best combination (MobileNet-SVM RBF) is highlighted in green.

**Table 3:** Accuracy, Precision, F1-Score, and Recall obtained through the classification of extracted features with CNNs architectures.

**Extractors**	**Classifiers**	**Accuracy**	**Precision**	**F1 Score**	**Recall**
DenseNet121	Naive Bayes	72.78 ± 1.88	80.75 ± 2.42	64.94 ± 3.36	65.57 ± 2.48
	MLP	75.78 ± 2.37	79.59 ± 4.55	71.66 ± 4.66	71.51 ± 3.88
	kNN	87.44 ± 2.44	89.13 ± 2.56	86.12 ± 2.86	84.90 ± 2.93
	RF	85.22 ± 2.81	87.35 ± 3.09	83.44 ± 3.35	82.09 ± 3.35
	SVM Linear	89.67 ± 3.65	90.21 ± 4.20	88.89 ± 3.90	88.17 ± 3.85
	SVM Polynomial	38.89 ± 0.00	19.44 ± 0.00	28.00 ± 0.00	50.00 ± 0.00
	**SVM RBF**	**89.78** **±** **3.51**	**90.32** **±** **4.05**	**89.01** **±** **3.74**	**88.31** **±** **3.69**
DenseNet169	Naive Bayes	71.00 ± 4.26	77.11 ± 7.90	62.22 ± 6.80	63.55 ± 5.00
	MLP	72.56 ± 6.05	77.58 ± 8.26	65.86 ± 8.90	66.64 ± 7.39
	kNN	87.22 ± 2.78	88.30 ± 3.10	86.04 ± 3.22	85.13 ± 3.38
	RF	86.56 ± 3.67	89.05 ± 3.23	84.85 ± 4.48	83.44 ± 4.51
	SVM Linear	89.11 ± 5.01	90.23 ± 5.05	88.05 ± 5.63	87.09 ± 5.84
	SVM Polynomial	38.89 ± 0.00	19.44 ± 0.00	28.00 ± 0.00	50.00 ± 0.00
	**SVM RBF**	**91.33** **±** **3.36**	**92.26** **±** **3.50**	**90.60** **±** **3.69**	**89.69** **±** **3.82**
DenseNet201	Naive Bayes	70.78 ± 2.11	83.49 ± 1.06	60.22 ± 4.06	62.48 ± 2.78
	MLP	76.56 ± 4.83	81.34 ± 5.81	71.74 ± 7.26	71.42 ± 6.13
	kNN	85.56 ± 4.04	86.63 ± 4.11	84.06 ± 4.61	82.99 ± 4.70
	RF	84.56 ± 4.75	86.57 ± 4.47	82.58 ± 5.79	81.44 ± 5.84
	SVM Linear	89.56 ± 2.95	89.89 ± 2.79	88.77 ± 3.34	88.29 ± 3.79
	SVM Polynomial	43.44 ± 9.11	26.70 ± 18.37	30.29 ± 4.63	50.14 ± 0.43
	**SVM RBF**	**90.00** **±** **2.58**	**90.30** **±** **2.04**	**89.25** **±** **3.00**	**88.86** **±** **3.60**
Inception ResNet V2	Naive Bayes	67.44 ± 5.86	65.54 ± 6.93	63.26 ± 6.85	63.18 ± 6.42
	MLP	72.22 ± 5.07	72.82 ± 7.24	68.77 ± 5.37	68.49 ± 5.22
	kNN	84.44 ± 4.22	85.55 ± 4.14	82.74 ± 5.04	81.77 ± 5.11
	RF	83.00 ± 4.25	85.07 ± 4.02	80.72 ± 5.23	79.55 ± 5.26
	SVM Linear	87.56 ± 2.52	88.16 ± 2.81	86.51 ± 2.76	85.66 ± 2.76
	SVM Polynomial	38.89 ± 0.00	19.44 ± 0.00	28.00 ± 0.00	50.00 ± 0.00
	**SVM RBF**	**87.56** **±** **2.71**	**88.37** **±** **3.38**	**86.48** **±** **2.92**	**85.51** **±** **2.79**
Inception V3	Naive Bayes	67.44 ± 5.94	67.49 ± 10.14	59.19 ± 8.36	60.58 ± 6.71
	MLP	74.89 ± 5.02	79.19 ± 4.33	69.57 ± 9.75	70.26 ± 7.44
	kNN	87.33 ± 4.16	89.20 ± 4.62	85.97 ± 4.59	84.70 ± 4.67
	RF	87.33 ± 3.95	90.11 ± 3.74	85.71 ± 4.64	84.18 ± 4.68
	**SVM Linear**	**89.56** **±** **2.23**	**90.87** **±** **1.93**	**88.56** **±** **2.60**	**87.51** **±** **2.98**
	SVM Polynomial	38.89 ± 0.00	19.44 ± 0.00	28.00 ± 0.00	50.00 ± 0.00
	SVM RBF	89.44 ± 2.24	90.87 ± 1.86	88.42 ± 2.61	87.31 ± 3.01
MobileNet	Naive Bayes	73.33 ± 4.87	74.70 ± 5.26	71.67 ± 5.27	71.60 ± 5.08
	MLP	86.89 ± 3.47	88.41 ± 2.70	86.55 ± 3.60	86.70 ± 3.44
	kNN	92.78 ± 1.88	93.01 ± 1.84	92.65 ± 1.93	92.53 ± 2.06
	RF	87.00 ± 3.62	90.03 ± 2.74	86.20 ± 3.99	85.50 ± 4.02
	SVM Linear	93.00 ± 2.33	93.16 ± 2.38	92.88 ± 2.38	92.73 ± 2.39
	SVM Polynomial	93.89 ± 2.59	94.16 ± 2.64	93.78 ± 2.64	93.60 ± 2.66
	**SVM RBF**	**95.33** **±** **1.47**	**95.68** **±** **1.31**	**95.24** **±** **1.52**	**95.00** **±** **1.63**
NASNetLarge	Naive Bayes	64.00 ± 2.73	69.64 ± 11.36	48.76 ± 3.90	54.60 ± 2.81
	MLP	72.56 ± 4.79	77.21 ± 6.50	67.62 ± 8.29	68.92 ± 6.62
	kNN	87.56 ± 4.30	88.54 ± 4.70	86.41 ± 4.67	85.40 ± 4.69
	RF	86.33± 2.33	88.17 ± 2.31	84.80 ± 2.82	83.52 ± 2.94
	SVM Linear	87.56 ± 3.10	88.61 ± 3.55	86.41 ± 3.59	85.45 ± 3.55
	SVM Polynomial	43.33 ± 8.89	21.67 ± 4.44	29.99 ± 3.97	50.00 ± 0.00
	**SVM RBF**	**90.33** **±** **2.72**	**91.70** **±** **3.29**	**89.47** **±** **2.95**	**88.35** **±** **2.88**
NASNetMobile	Naive Bayes	71.58 ± 3.23	71.50 ± 3.73	69.66 ± 3.51	69.39 ± 3.38
	MLP	86.74 ± 5.14	87.77 ± 4.81	86.12 ± 5.57	85.99 ± 5.46
	kNN	90.53 ± 2.58	91.35 ± 2.58	90.08 ± 2.76	89.47 ± 2.84
	RF	86.00 ± 2.54	86.92 ± 2.34	85.23 ± 2.87	84.64 ± 2.92
	SVM Linear	93.89 ± 3.04	94.56 ± 2.89	93.63 ± 3.19	93.16 ± 3.42
	SVM Polynomial	57.89 ± 0.00	28.95 ± 0.00	36.67 ± 0.00	50.00 ± 0.00
	**SVM RBF**	**92.74** **±** **2.72**	**93.32** **±** **2.74**	**92.44** **±** **2.87**	**91.95** **±** **2.94**

*The bold values are mean and standard deviation, respectively*.

**Table 4:** Continuation of Table 3.

**Extractors**	**Classifiers**	**Accuracy**	**Precision**	**F1 Score**	**Recall**
ResNet50	Naive Bayes	63.16 ± 0.00	31.58 ± 0.00	38.71 ± 0.00	50.00 ± 0.00
	MLP	66.32 ± 1.94	82.62 ± 0.68	47.17 ± 4.62	54.29 ± 2.63
	**kNN**	**87.68** **±** **3.40**	**89.01** **±** **3.97**	**86.07** **±** **3.88**	**84.71** **±** **4.00**
	RF	85.26 ± 4.16	86.81 ± 4.03	82.97 ± 5.35	81.61 ± 5.45
	SVM Linear	81.47 ± 2.50	80.59 ± 1.95	80.52 ± 2.28	81.40 ± 1.83
	SVM Polynomial	36.84 ± 0.00	18.42 ± 0.00	26.92 ± 0.00	50.00 ± 0.00
	SVM RBF	79.16 ± 1.87	77.95 ± 1.97	77.36 ± 2.15	77.25 ± 2.45
VGG16	Naive Bayes	64.00 ± 2.09	77.81 ± 4.35	51.25 ± 3.95	57.39 ± 2.46
	MLP	81.26 ± 4.34	82.14 ± 4.67	80.15 ± 4.66	79.66 ± 4.72
	kNN	90.84 ± 1.94	91.54 ± 1.89	90.44 ± 2.08	89.94 ± 2.29
	RF	87.79 ± 1.50	89.65 ± 1.70	87.00 ± 1.65	86.08 ± 1.75
	SVM Linear	86.63 ± 3.71	87.01 ± 3.67	86.16 ± 3.85	86.07 ± 3.84
	SVM Polynomial	57.89 ± 0.00	28.95 ± 0.00	36.67 ± 0.00	50.00 ± 0.00
	**SVM RBF**	**93.37** **±** **2.45**	**94.00** **±** **2.16**	**93.08** **±** **2.60**	**92.64** **±** **2.86**
VGG19	Naive Bayes	65.11 ± 1.59	77.94 ± 7.05	49.10 ± 2.96	55.40 ± 1.79
	MLP	78.56 ± 5.07	81.53 ± 5.42	74.76 ± 6.80	73.88 ± 6.19
	kNN	91.11 ± 2.72	91.53 ± 3.04	90.51 ± 2.81	89.97 ± 2.66
	RF	86.67 ± 3.51	88.90 ± 3.10	85.04 ± 4.27	83.69 ± 4.35
	SVM Linear	85.44 ± 3.86	84.92 ± 4.11	84.63 ± 4.04	84.56 ± 4.05
	SVM Polynomial	38.89 ± 0.00	19.44 ± 0.00	28.00 ± 0.00	50.00 ± 0.00
	**SVM RBF**	**91.89** **±** **2.98**	**92.56** **±** **2.89**	**91.24** **±** **3.30**	**90.45** **±** **3.51**
Xception	Naive Bayes	66.00 ± 4.45	63.98 ± 5.23	63.02 ± 4.86	62.88 ± 4.76
	MLP	74.78 ± 4.07	75.45 ± 4.92	71.32 ± 5.30	70.95 ± 5.15
	kNN	88.11 ± 2.54	88.89 ± 3.24	87.11 ± 2.73	86.22 ± 2.72
	RF	88.78 ± 2.96	90.36 ± 3.26	87.66 ± 3.32	86.45 ± 3.31
	SVM Linear	90.78 ± 2.17	91.47 ± 2.52	90.06 ± 2.35	89.23 ± 2.38
	SVM Polynomial	45.56 ± 10.18	22.78 ± 5.09	30.98 ± 4.55	50.00 ± 0.00
	**SVM RBF**	**92.56** **±** **2.11**	**93.35** **±** **2.14**	**91.97** **±** **2.31**	**91.16** **±** **2.49**

*The bold values are mean and standard deviation, respectively. Accuracy, Precision, F1-Score, and Recall obtained through the classification of extracted features*.

Figure 5 compares the best combination of the traditional methods and the CNN architectures. The features extracted by the CNN-MobileNet and classified by SVM RBF achieved an accuracy 8% higher than the features extracted by the LBP and classified by RF. Also, the standard deviation for MobileNet+SVM RBF is lower, contributing to greater reliability for the system. Furthermore, even though the combination LBP+RF has an accuracy of 87%, its recall is only 84%, while the combination MobileNet+SVM RBF has accuracy and recall of 95%.

The results show that the number of features, according to Table 1, indicate that traditional feature extraction methods have a low representative potential. On the other hand, the feature extraction through CNN can extract more information, and this contributed to improving the classification results. Besides, tests in other bands with lower frequency channels, such as F5, TP7, PO7, and O1, did not reach metrics with values higher than 95.33%, as we achieved with the channels proposed in this work.

Acharya et al. (2012), approach, the 4 HOS features were not able to detect the most relevant features for class distinction, reaching an average accuracy of 91.7%. While the works of Ehlers et al. (1998), Kannathal et al. (2005), and Rachman et al. (2016) used statistical analysis of EEG signals. However, the use of the average value as a descriptor of the samples made the classification sensitive to extreme values. In addition, the use of descriptors with a fixed range of analysis makes it difficult to generalize unknown samples. All of these studies presented an average of <90%. Table 5 gives a summary of the characteristics of these approaches.

**Table 5:** Summary and statistical comparison to other methods to classification alcoholism and healthy patients.

**Author**	**Data**	**Channels**	**Subjects**	**Features/Method**	**Classification of features**	**Performance metrics**			
						**Acc**	**Precision**	**F1 Score**	**Recall**
Proposed method	UCI KDD	11	20	CNN as feature extractor	MobileNet + SVM RBF	95.33 ± 1.47	95.68 ± 1.31	95.25 ± 1.52	95.00 ± 1.63
Acharya et al. (2012)	Bern Barcelona database	2	3000	Entropy, 4 HOS features, Largest Lyapunov Entropy	SVM (linear, polynomial, and RBF kernels)	91.7	93.9	–	90
Rachman et al. (2016)	UCI KDD	64	77	Daubechies wavelet family	Maximum, minimum, average and standard	85	–	–	100
Mumtaz et al. (2016)	University Malaya Medical Center	19	45	Power Spectral Density (PSD)	Logistic Regression	89.5	88.5	91	90
Ehlers et al. (1998)	University of California	1	32	CD	Discriminant analysis	88	–	–	–
Kannathal et al. (2005)	UCI KDD	60	30	CD, LLE, entropy, H	Filter by unique ranges	90	–	–	–
Faust et al. (2013)	UCI KDD	61	60	HOS cumulants	FSC	92.4	91.1	–	94.9
Patidar et al. (2017)	UCI KDD	64	122	Tunable Q-wavelet transform	Correntropy, Low-frequency(LF)-rhythms based statistical features	97.02	–	–	96.53

The work of Faust et al. (2013) analyzed the signals using a non-linear approach. Accumulating the HOS characteristics, and combined the extractions with a Fuzzy Sugeno Classifier reached 92.4%. However, an approach using fuzzy classification imposes the need for prior knowledge of the data set for method calibration, and this makes the approach semi-automatic. Our approach does not require previous knowledge of EEG signals since the extraction models use the transfer learning techniques for feature extraction to achieve promising results.

We see in Table 6 the results obtained by the methods proposed by Acharya et al. (2012) and Mumtaz et al. (2016). We obtain the results using extractors and classifiers proposed with the same parameters of cross-validation and dataset that we used in our method. Thus, we show the efficiency of our method within the set of EEG channels that we chose in our work. Both compared to a method that uses non-linear features and against a method that uses features in the frequency domain, respectively.

**Table 6:** Accuracy, Precision, F1-Score, and Recall obtained by Acharya et al. (2012) and Mumtaz et al. (2016) proposed methods.

**Method**	**Classifiers**	**Accuracy**	**Precision**	**F1 Score**	**Recall**
Acharya et al. (2012)	**SVM Linear**	**50.67** **±** **19.05**	**49.24** **±** **23.82**	**46.67** **±** **20.73**	**50.67** **±** **19.05**
	SVM Polynomial 1	48.5 ± 11.51	46.49 ± 20.88	41.04 ± 12.04	48.5 ± 11.51
	SVM Polynomial 2	49.83 ± 4.61	44.95 ± 26.64	35.34 ± 5.66	49.83 ± 4.61
	SVM Polynomial 3	49.67 ± 2.33	34.91 ± 21.47	34.01 ± 2.69	49.67 ± 2.33
	SVM RBF	50.17 ± 2.42	37.54 ± 21.67	34.76 ± 3.50	50.17 ± 2.42
Mumtaz et al. (2016)	**Logistic Regression**	**58.00** **±** **10.18**	**60.10** **±** **10.64**	**54.92** **±** **12.18**	**58.00** **±** **10.18**

*The bold values are mean and standard deviation, respectively*.

Finally, the proposed approach presented superior results to all the methods considered in this study. Our approach achieved accuracy values equivalent to the work of Mumtaz et al. (2016), considering the standard deviation. However, our approach innovated by applying a 2D analysis of the EGG signal, which allowed the application of CV techniques to overcome the problem. Table 5 presents the results of the proposed approach of this paper compared with other works available in the literature.

## 7. Conclusions and Future Works

In this work, we proposed a new method to detect a predisposition to alcoholism from image-transformed EEG signals using traditional and deep feature extractors. We used the Learning Transfer method to extract deep image characteristics and consolidated ML methods to classify EGG signals between alcoholism and normal.

From the results presented, we can see that the CNN architectures extracted more relevant features from the samples, since the best values of Accuracy 95.33%, Precision 95.68%, F1-Score 95.24%, and Recall 95.00% were obtained in the MobileNet-SVM RBF combination. The best combination for classic extractors was LBP-RF reaching 87.33, 89.08, 85.96, and 84.75% for the same metrics.

For future work, we will apply the Principal Components Analysis (PCA) algorithm to select the most significant channels after preprocessing in order to highlight the differences between the features of each class. Another possibility is the application of fuzzy logic as a method of filtering EGG signals after preprocessing, as well as the application of mathematical morphology to highlight the differences between image textures after 1D to 2D transformation.

## Data Availability Statement

Publicly available datasets were analyzed in this study. This data can be found here: https://kdd.ics.uci.edu/databases/eeg/eeg.data.html.

## Author Contributions

HZ designed and supervises all the aspects of the study implementation and drafted the manuscript. FS and AM performed the experiments based on the 2-dimensional EEG signal approach. EO applied the traditional computer vision methods for comparison with existing techniques and revised the manuscript. PR oriented the development of this work. All authors contributed to the article and approved the submitted version.

## Conflict of Interest

The authors declare that the research was conducted in the absence of any commercial or financial relationships that could be construed as a potential conflict of interest.
